# Supplementary material for: Development of a clinical prediction score for Ebola virus disease screening at triage centers in the Democratic Republic of the Congo
Source: PLOS Glob Public Health. 2024 Aug 26;4(8):e0003583. doi: 10.1371/journal.pgph.0003583 (PMC11346649; doi:10.1371/journal.pgph.0003583)
Supplement: Attachment — Submitted filename: Response to reviewers.docx [file pgph.0003583.s002.docx]

Exp: YANGO WA YANGO Jepsy Grace

Institut National de Recherche Biomédicale

Adress : 8, Ngombi, Ngaliema, Kinshasa, DR. Congo

Email: [jepsyango@gmail.com](mailto:jepsyango@gmail.com)

Tel: +243815205343

June 14. 2024

Maria del Pilar Fernandez

Academic Editor

PLOS Global Public Health

Dear Editor Maria del Pilar Fernandez,

Thank you for your comprehensive review and constructive feedback on our manuscript entitled “Development of a Clinical Prediction Score for Ebola Virus Disease Screening at Triage Centers in the Democratic Republic of Congo.” We are grateful for the opportunity to refine our submission and address the concerns raised by the reviewers.

In response, we have carefully revised our manuscript to enhance clarity, precision, and adherence to the latest scientific standards. Below, we summarize the primary revisions and address the specific points highlighted during the review process:

1. Taxonomic Clarifications: We have updated the taxonomy of the Ebola virus in accordance with the latest ICTV guidelines, ensuring the manuscript reflects precise and accurate scientific terminology.
2. Methodological Details: We have provided additional clarifications and expansions in the methods section, particularly detailing the triage and testing protocols used at the ETUs.
3. Statistical Analysis Enhancements: The statistical analysis section has been fortified to offer more detailed support for the validity of our findings, ensuring our data interpretation is thorough and transparent.
4. Response to Reviewer Queries: We have meticulously addressed each point raised by the reviewers, making appropriate adjustments to the manuscript. The specific responses are detailed in the attached rebuttal letter.

We have uploaded the following documents for your review:

- The clean version of the manuscript, file labelled “Manuscript”
- The track changes version of the manuscript, file labelled “Revised Manuscript with Track Changes”
- The rebuttal letter addressing the editorial and referees’ comments, file labelled “Response to Reviewers”

We believe these enhancements significantly strengthen our manuscript, making the findings more compelling and our conclusions more definitive. We hope the revised version meets the esteemed standards of PLOS Global Public Health and look forward to the possibility of our work contributing valuably to the field.

Thank you for considering our revised manuscript. We eagerly anticipate your feedback and hope for a favorable resolution.

Kind regards,

Jepsy Grace YANGO WA YANGO

Institut National de Recherche Biomédicale

**Reviewer #1:**

Dear Reviewer, #1,

We appreciate your insightful comments and suggestions, which have greatly improved our manuscript.

Below are our detailed responses to each of your points:

Q1. The authors make minor errors in referring to the taxonomy of Ebola virus. For example, at line 71 they state ‘Different species of Ebola virus exist with Zaire ebolavirus […]’. This is incorrect. Ebola virus (EBOV) is the sole member of the species Orthoebolavirus zairense. Other species within the genus Orthoebolavirus do indeed exist (and confusingly these are properly referred to as ‘ebolaviruses’ – lower case and not italicized) but they are distinct viruses (e.g., Sudan virus (SUDV) member of species Orthoebolavirus sudanense and the etiological agent of Sudan virus disease (SVD)).

**Response**: We have corrected the taxonomy of Ebola virus to accurately reflect the current classification. The revised text specifies that EBOV is the sole member of the species Orthoebolavirus zairense. The updated sentence reads: "Ebola virus disease (EVD) is caused by the Ebola virus (EBOV), which is now classified under the species *Orthoebolavirus zairense*, a member of the Filoviridae family in the genus *Orthoebolavirus*. "

Q2. At line 333, the authors use the term ‘Ebola strain’; this too is incorrect. It should say ‘Ebola variant’ as that correctly references the different Ebola viruses that have spilled over in DRC West Africa etc. Regarding correct Ebola virus nomenclature taxonomy etc.

I refer the authors to the ICTV

website: <https://ictv.global/report/chapter/filoviridae/filoviridae/orthoebolavirus> and a very helpful article by Kuhn et al. (<https://www.ncbi.nlm.nih.gov/pmc/articles/PMC6637750/>). While these are technical points, it is beneficial to readers and to the filoviral literature as a whole when precise correct terminology is used.

**Response:** We have replaced the term ‘Ebola strain’ with ‘Ebola variant’ throughout the manuscript. This correction ensures precise and accurate terminology in line with the ICTV guidelines and Kuhn et al. (2019).

Q3. The authors repeatedly state that the EVD epidemic referenced in their study was the 10th such outbreak in DRC (e.g., lines 78, 116-117, etc.). They mistakenly include (as many others have done including MSF) the 2012 outbreak of Bundibugyo virus (BDBV), a member of the species Orthoebolavirus bundibugyoense and the etiological agent of Bundibugyo virus disease (BVD). While certainly a very similar virus that causes similar disease to EBOV in humans, it is not the same, and the conclusions of this study should not necessarily be extrapolated to BDBV (or any of the other ebolaviruses). I think it would be best to avoid continually referring to the epidemic at hand with any reference to its number (9th, 10th whatever) to avoid confusion. Again, see the taxonomy discussion above.

**Response:** We have removed references to the specific number of the EVD outbreak and refer to the outbreak period to avoid confusion. We now refer to it simply as the EVD 2018-2020 outbreak in the DRC.

Q4. Lines 84-85 ‘Following the WHO case definition, symptomatic patients were admitted to the Ebola treatment unit (ETU) and subjected to a PCR test (GeneXpert) for the diagnosis of EVD’. The chronology here seems incorrect. Actual admission to the ETU should be dependent upon a positive PCR. A similar statement is present at lines 355-357. During the West Africa epidemic, most ETUs had triage areas where patients were stratified for PCR testing before being definitively admitted to the ETU following a positive test. Perhaps it worked differently in Beni, but the authors should clarify the triage and admission process since this is integral to understanding the utility of their tool and the paper as a whole.

**Response:** We have clarified the triage and admission process in Beni. The revised text now accurately reflects that the admission of symptomatic patients to the ETUs was based on a positive RT-PCR test.

The updated text reads: Initially, patients were stratified in triage areas according to the case definition, and only those testing positive with RT-PCR were definitively admitted to the ETU for further treatment. Following the WHO case definition, symptomatic patients were admitted to Ebola treatment units (ETUs) based on a positive reverse transcription polymerase chain reaction (RT-PCR) test (GeneXpert).

Q5. The authors should add further discussion regarding the risks inherent to using a tool such as this for triage decision making. A tool that is too sensitive will suggest that many patients without EVD be admitted and increase the risk of nosocomial infection by exposure to those who are truly infected within the ETU. A tool that is too specific will inevitably release truly infected individuals back to the community with a presumptive negative EVD diagnosis and thus risk starting new chains of unrecognized transmission outside of the ETU. Thus, the stakes are so high with EVD that NAAT, rapid antigen testing, or other molecular methods are the only way to effectively manage an outbreak. The authors do generally acknowledge this but should more specifically and definitively do so.

**Response:** We have expanded the discussion to more specifically address the risks associated with using the clinical prediction score (CPS) for triage decision making. The revised text now emphasizes the importance of balancing sensitivity and specificity to avoid nosocomial infections and missed diagnoses, and highlights the necessity of using molecular methods for definitive outbreak management.

Q6. Also, they mention the need for RDTs but make no reference to the RDTs that are currently available (e.g., OraQuick). Some explanation for this should be provided.

**Response:** We have added a reference to the currently available RDTs, including Mukadi-Bamuleka D et al. (2023), available on : <https://pubmed.ncbi.nlm.nih.gov/37084479/> the conclusion of this study was : None of the RDTs evaluated met the WHO's "desired or acceptable levels" for sensitivity, but all met the "desired level" for specificity. Despite this, the QuickNavi-Ebola™ and OraQuick® Ebola Rapid Antigen Test showed the most favorable profiles and could be used as frontline tests for triaging suspected cases while awaiting RT-qPCR confirmatory testing.

**Reviewer #2,**

Dear Reviewer, #2,

We appreciate your constructive feedback and detailed suggestions. Below, we provide our responses to each comment and detail the revisions made to address them:

I. **Introduction**

Q1. Use the ongoing classification for Filoviridae. (https://pubmed.ncbi.nlm.nih.gov/37537381/)

Response: We have updated the manuscript to use the current classification for Filoviridae. The text now accurately reflects the latest classification standards.

Q2. Lines 75-76 are not well reflected as Sudan outbreak is overseen.

Response: We have revised the text to correctly reflect the Sudan outbreak and ensure accurate representation.

Q3. Lines 76-77: West African outbreak started in 2013. Correct.

Response: We have corrected the text to indicate that the West African outbreak started in 2013.

Q4. Lines 81-82: the sentence could be more precise, for example, the MoH of the DRC…

Response: The text has been revised for clarity. The revised sentence now reads: "The Ministry of Health (MoH) of the Democratic Republic of Congo (DRC), with support from the World Health Organization (WHO) and other partners, led the outbreak response."

Q5. Line 85: correct RT-PCR instead of PCR throughout the manuscript. If using abbreviations, first write them in full at the first citation then abbreviate for the rest…

Response: We have ensured that RT-PCR is correctly used and abbreviated appropriately throughout the manuscript.

Q6. Lines 86-92: Assuming that the response was hampered by poor access to the GX is not correct. On the other hand, the GX reduced the turnaround time, improved the diagnostic, and was the key tool in the success of > 200,000 samples tested throughout that outbreak. (https://pubmed.ncbi.nlm.nih.gov/36573642/).

Response: We would like to clarify that our remark on the challenges faced in the Ebola response did not intend to question the effectiveness of the GeneXpert system, which indeed reduced turnaround times, improved diagnostics, and was crucial for testing over 200,000 samples during the outbreak. Accessibility to the GeneXpert system was hampered not by a shortage of machines but by geographical barriers—such as significant distances between villages and equipped laboratories—and security issues, with several areas being under the control of rebel forces.

These factors severely restricted the ability to effectively deploy this technology in some of the hardest-hit regions. Our observation focused on specific logistical challenges in deploying technology and receiving lab results promptly in complex geographic and security contexts.

This study (<https://pubmed.ncbi.nlm.nih.gov/36573642/> ) states that during the 10th EVD outbreak, field laboratories faced tremendous challenges. The seven main challenges were:

1. Activities interruption after attacks on response teams;
2. Movement of contacts and suspected and confirmed case-patients resulting in further spread of the disease;
3. Slow resumption of activities after security incidents;
4. Evacuation of response staff out of outbreak areas during insecurity events;
5. Delayed implementation of activities;
6. Disruption to refilling laboratory supplies and fuel;
7. Delayed sample transportation to the laboratory (especially for the sequencing unit).

The challenges mentioned in this publication align with the difficulties outlined in our study regarding the identification (lab diagnostic), isolation, and rapid treatment of cases during an epidemic.

Q7. Lines 87-89 should be revised in line with the new statement authors will produce.

Response: The text has been revised accordingly.

Q8. Lines 90-92: better to say discriminate rather than differentiate. What means the term “other conditions”? Rephrase.

Response: We have replaced “differentiate” with “discriminate” and clarified “other conditions” to “other illnesses”. The revised sentence reads: "The WHO case definition of active EVD had a sensitivity of 81.5% and a low specificity of 35.7%, leading to a poor capacity to discriminate EVD from other illnesses."

Q9. Lines 93-103 should be rephrased to clearly show the junction authors want to do with the study objective.

Response: We have rephrased these lines for clarity, ensuring they clearly articulate the connection to the study's objective.

Q10. Sentence containing the objective is very long and difficult to read: “EVD outbreaks” and “at the triage point” are stated twice.

Response: The sentence has been revised for clarity and conciseness.

Q11. Lines 111-112: I do not see its usefulness there.

Response: We have removed the sentence.

II. **Methods**

Q1. Methods, not method.

Response: Corrected to "Methods".

Q2. Study settings should be rewritten to meet the Editor’s criteria. Not well written.

Response: The study settings section has been rewritten for clarity and alignment with the journal’s criteria.

Q3. Inclusion and exclusion criteria: you only considered data from one transit center in Beni, whereas there were others in the regions…Is it a personal choice or a lack of data to exploit?

Response: The focus on the Beni Transit Center was due to a lack of exploitable data from other centers.

Q4. EVD suspect-cases could also come from active surveillance in the community, investigation.

Response: We have included this information in the inclusion criteria.

Q5. Lines 133-134: correct the structure of the sentence.

Response: The sentence has been corrected.

Q6. Lines 165-166: Add the brand/company, City, country for R.

Response: We have added this information.

Q7. Lines 168-170: drop one “the Tshomba AO et al” and rephrase.

Response: The text has been rephrased.

Q8. Lines 170: The objective “was to evaluate” instead of “is to evaluate”.

Response: The objective has been corrected.

Q9. Can authors clearly state what positive LR and negative LR really mean in the interpretation as it is not clearly defined.

Response: We have added definitions for positive and negative likelihood ratios:

- Positive Likelihood Ratio (LR+) indicates how much more likely a positive test result is to occur in someone with the disease compared to someone without the disease. An LR+ greater than 1 suggests that the symptom is associated with the disease.
- LR+ = Sensitivity/(1-Specificity)
- Negative Likelihood Ratio (LR-) indicates how much less likely a negative test result is to occur in someone with the disease compared to someone without the disease. An LR- less than 1 suggests that the absence of the symptom is associated with not having the disease.
- LR- = (1-Sensitivity)/Specificity

Q10. Lines 183-184: specify comité d’éthique de l’Ecole de Santé publique de Kinshasa in French or English.

Response: the text has been revised and updated in English

III. **Results**

Q1. Results should be presented as one body with reference to different objects (tables and figures) following a logical pathway. Here it is just a superposition of figures without clear logical course.

Response: The results have been revised to present them as one cohesive body with logical references to tables and figures.

Q2. Add a flow diagram if possible

Response: A flow diagram has been added.

Q3. Lines 190-192 should be rephrased to allow easy reading.

Response: These lines have been rephrased for clarity.

Q4. Decimals should be consistently written as per the journal’s requirements: use comma or point, not both in the same manuscript. Lines 196-197: the sentence looks incomplete.

Response: Decimals have been standardized, and the incomplete sentence has been corrected.

Q5. Should tables and figures go along with the manuscript or be presented in separate documents?

Response: Revised to ensure tables and figures are appropriately placed.

According to PLos requirements, tables and figures can be included with the manuscript or separated from it.

Q6. Figure one could be consistently commented instead of a brief description.

Response: Figure one has been given a more comprehensive comment.

Q7. Table 4 is poorly commented, not reflecting the content and the interpretation we would expect for such a data.

Response: Table 4 has been thoroughly commented to reflect its content and interpretation better.

IV. **Discussion**

Q1. The discussion section can be presented as one body text. Can drop most subheadings in the section. The limitations and strengths can be read without subheadings.

Response: The discussion has been revised to present it as one body text without most subheadings.

Q2. Lines 251-252: It is not clear to me whether the score clearly helped to discriminate EVD from other illness. The performance the ROC is purely theoretical, and I did not see how the discrimination was clearly done between EVD and other illness. Otherwise, authors can lay more on that.

Response: The ROC curve provides a theoretical measure of the score’s performance at different possible cut-offs. In-practice, discrimination happens at the individual thresholds presented in Table 4, leading to the decision tree in Figure 3.

The practical application of CPS is supported by the specificity of the symptoms chosen, the epidemiological context, and validation against external datasets. This combined approach ensures that the score not only theoretically but also practically helps in discriminating EVD from other illnesses, particularly in outbreak settings.

However, like all other existing prediction tools for Ebola disease, this score is imperfect in its ability to discriminate between Ebola and differential diagnoses, and should therefore only be used for the intended triaging purposes as shown in Figure 3 and explained in the discussion.

Q3. I would like to read more about the possible combination of the score with Ebola rapid diagnostic tests to see whether the performance could be increased.

Response: The combination of clinical predictors with RDTs could provide a more robust and efficient diagnostic tool, reducing the time to diagnosis and improving the accuracy of distinguishing EVD from other febrile illnesses. Our future studies will focus on developing and validating this integrated approach.

Q4. What will be the authors’ advice on pauci-symptomatic cases if the score has to be used? What comment can drawn from this?

Response: Future studies will focus on refining the CPS to better capture pauci-symptomatic presentations. This may involve integrating more sensitive markers and utilizing machine learning algorithms to analyze subtle symptom patterns and epidemiological data. Additionally, combining the score with rapid diagnostic tests (RDTs) will be a priority to enhance overall diagnostic performance.

Q5. Lines 285-294: From the interpretation of the score, I do understand that any score presented will call for at least a 21-day follow-up. In this condition, I’m wondering if the score really helps discriminating EVD from other illnesses as stated in the manuscript?

Response: It is important to clarify that the score's purpose is to aid in the initial risk stratification and decision-making process, rather than serving as a definitive diagnosis that negates the need for follow-up.

Q6. Lines 295-305: I do not see in the text what the risk categorization brings in practices regarding case management at the triage unit.

The risk categorization is directly linked to the subsequent actions taken. This is now expanded upon in the manuscript.

Q7. The algorithm should be reinforced with a decisional tree clarifying EVD suspects' referral process or decision taken for care management.

Response: Response: "The figure (algorithm) used is in essence a decision tree, as we have incorporated the relevant actions."

Q8. Lines 315-319: Can authors take the responsibility for discharging EVD suspects categorized as low or medium risk without two successive RT-PCR results within 48-72 hours? If yes, this should be stated in the manuscript. If not, authors should correct lines 311-319.

Response: The algorithm proposed in the study suggests that suspected EVD cases categorized as high or medium risk should be isolated, while low-risk cases undergo follow-up within 21 days. Low-risk patients can be managed with routine care and monitored for symptom development. However, ideally, they should not be discharged without at least one RT-PCR test confirming they are negative for EVD. If resources allow, a follow-up RT-PCR test should be performed at the earliest availability to ensure patient safety and public health security.

It is common knowledge that in an ideal world everyone can be tested with RT-PCR in a timely manner and such risk categorization is mostly redundant.
However, in settings with challenges and/or insufficient capacity, difficult choices must be made based on the potential harm they may induce both on the presenting patients and the community. If everyone presenting would be isolated at the facility for 21 days due to a lack of sufficiently available RT-PCR tests, this would inevitably lead to a spike of distrust in the community and later presentation of true cases.

Consequently, it’s not up to us to say that patients should be discharged without negative RT-PCR result as this should never be the default decision, but this option should not be dismissed firsthand either.

Q9. Can authors clearly assess the easiness of the tool to be used in the field conditions by health workers without mistaking or being overloaded, especially if more suspect-cases are received per day? Evaluating the time taken for consultation and experimentation of the score will be a key point to really know if this tool will be time-saving or cumbersome for healthcare workers in the field.

Response: To address the question regarding the ease of use of the CPS by HCWs in field conditions, especially when managing a high volume of suspect cases:

- The CPS is designed with simplicity and clarity in mind, using straightforward criteria such as specific symptoms and epidemiological factors. This reduces the risk of errors and ensures that healthcare workers can quickly and accurately assess patients.
- Minimal training is required for HCWs to understand and implement the score. Training can be incorporated into existing HCW training programs, emphasizing the key symptoms and epidemiological factors to look for.
- The score can be easily implemented in field settings, using paper forms or digital tools to record patient information and calculate the score.
- The time taken to apply the clinical prediction score during patient consultations is minimal. HCWs can quickly assess the presence of the seven key factors (sore throat, asthenia, conjunctivitis, bleeding gums, hematemesis, contact with a sick person, and contact with a traditional healer) without extensive diagnostic procedures.

Q10. Lines 330-331: I don’t understand what is written there.

Response: We have improved the text for clarity. The revised sentence reads: "Moreover, in scenarios where the number of suspected EVD cases exceeds the standardized capacity of healthcare facilities, the CPS proves to be an essential tool for triage. It allows HCWs to quickly assess and prioritize patients based on their risk level, ensuring that those who need immediate attention are isolated and tested promptly."

Q11. Can authors say if this tool can be equally used at the tail and/or at the beginning of an EVD outbreak? Can lay a little bit more on that in the discussion section.

Response: We emphasized that the CPS can be effectively applied at different stages of an EVD outbreak, ensuring continuous and effective disease management.

At the onset of an outbreak, the score helps rapidly identify and isolate high-risk individuals, preventing the spread of the disease. Early in an outbreak, with a high volume of suspected cases, the score aids in managing the surge by prioritizing those most likely to have EVD. Towards the end of an outbreak, when cases decrease, the score remains valuable for accurately identifying the few remaining cases, preventing a resurgence.

By being adaptable to different stages, the CPS provides a flexible and structured approach to managing EVD outbreaks, enhancing the ability of HCWs to respond promptly and effectively throughout the epidemic.

V. **Conclusion**

Q1. The conclusion varies from the study objective, can authors align this? The objective is stuck on the score and the conclusion focuses on RDTs…Not

consistent…

Response:

we rewrite the conclusion and improve based on the comment provided by the reviewer
